# Establishing Reference Values for Vaginal Vestibule Elastography in Healthy Postmenopausal Women

**DOI:** 10.7759/cureus.68294

**Published:** 2024-08-31

**Authors:** Nobuo Okui

**Affiliations:** 1 Dentistry, Kanagawa Dental University, Kanagawa, JPN; 2 Urogynecology, Yokosuka Urogynecology and Urology Clinic, Kanagawa, JPN

**Keywords:** real-time tissue elastography technology, gynecological conditions, healthy women, postmenopausal women, elastography

## Abstract

Objective

This study aims to establish normative elastography values for the vaginal vestibule in healthy postmenopausal women and assess the variability of these values with age and measurement time during the day.

Methods

The study included 111 women aged 40-90, excluding those with medical histories or treatments affecting vaginal health. Elastography measurements were taken twice daily, between 9-10 AM and 3-4 PM, using real-time tissue elastography technology. Statistical analysis evaluated the effects of age, body mass index (BMI), and diurnal variation on vaginal vestibule elasticity.

Results

A significant positive correlation between age and elastography values was found (p=8.36×10⁻⁷), with elastography values increasing by approximately 0.0040 units per year. The mean elastography value was 28.32% (SD=5.87%) in the morning and 28.10% (SD=5.90%) in the afternoon, with a statistically significant difference (p=0.016). BMI showed a weak negative correlation with elastography values (r=-0.2021, p=0.0334).

Conclusion

Establishing reference values for vaginal vestibule elastography provides a foundation for improved diagnostic accuracy and early detection of gynecological conditions. The findings support the use of elastography as a non-invasive, reliable diagnostic tool in clinical practice. Future research should validate these results across different age groups and in women with specific gynecological conditions to further solidify the clinical applicability of vaginal vestibule elastography.

## Introduction

Elastography of pelvic organs, including genital and urinary organs, has recently gained attention for the diagnosis and evaluation of conditions such as pelvic organ prolapse (POP) and vaginal atrophy [1-6]. Elastography technology is anticipated to be a noninvasive and rapid diagnostic tool because of its ability to quantitatively assess tissue elasticity. Despite its advantages, this technology has limitations, such as operator dependency and the qualitative nature of the data. Furthermore, there is a complete lack of data on normal elasticity values for the vaginal vestibule, which may affect the accuracy and reliability of diagnosis [7].

Previous studies have evaluated the vagina and pelvic floor muscles using elastography. However, none have aimed to establish normal values; instead, most studies have focused on research applications for specific diseases [1-6]. Disease diagnosis without clearly defining normal values may increase the risk of misdiagnosis due to ambiguous diagnostic criteria. Therefore, it is essential to clarify the normal elasticity values of the vaginal vestibule and understand how they fluctuate with age and measurement duration.

The objective of this study was to establish normal elastography values of the vaginal vestibule in healthy women aged 40-90 years. Specifically, we evaluated the variation in elasticity values due to age and the effect of the measurement time during the day. This study included 111 subjects, excluding those with conditions such as breast cancer treatment, uterine or ovarian surgery, bladder or urethral surgery, and hormone therapy. Measurements were taken twice, between 9-10 AM and 3-4 PM, and the obtained data were statistically analyzed.

This research will clarify the normal elasticity values for elastography of the vaginal vestibule, laying the foundation for future clinical studies. Establishing normal values is expected to improve the accuracy of abnormality detection and contribute to the early diagnosis of gynecological diseases and the formulation of appropriate treatment plans.

## Materials and methods

Study design and participants

This study was conducted at Yokosuka Urogynecology and Urology Clinic with approval from the Institutional Ethics Committee. The participants were female patients who underwent cervical cancer screening between January and June 30, 2023.

Sample size calculation

The sample size calculation was performed using an expected correlation coefficient of 0.44, a significance level of 0.05, and a power of 0.80, resulting in a minimum required sample size of 39 individuals.

Inclusion and exclusion criteria

Inclusion criteria were the patients' ability to understand and explain vaginal symptoms, as well as having a healthy vagina, external genitalia, bladder, and urethra, along with normal urinalysis and cervical smear results. Exclusion criteria included a history of various medical conditions and treatments, such as breast cancer treatment, hormone therapy, pelvic surgery (including uterine, ovarian, or bladder surgery), POP surgery, and POP (stage 3 or higher).

Participants were also excluded if they had conditions affecting the urinary system or pelvic area, including overactive bladder, urinary incontinence, interstitial cystitis, recurrent urinary tract infections, urinary retention, neurogenic bladder, bacterial vaginosis, fungal vaginitis, vulvodynia, pelvic pain syndrome, and pain in genitals or external genitalia upon palpation. Furthermore, individuals with collagen diseases (e.g., Sjögren's syndrome and systemic lupus erythematous) and congenital anomalies (ectopic urethral opening, vaginal agenesis, and Mayer-Rokitansky-Küster-Hauser syndrome) were excluded from the study. 

The use of medications that may affect vaginal health or elasticity was also an exclusion criterion. These medications included but were not limited to diuretics, antihypertensive drugs (e.g., angiotensin-converting-enzyme (ACE) inhibitors and beta-blockers), corticosteroids, immunosuppressants, chemotherapy agents, antidepressants (e.g., selective serotonin reuptake inhibitors (SSRIs) and tricyclics), antipsychotics, anticonvulsants, hormonal medications (e.g., oral contraceptives and hormone replacement therapy), and systemic antibiotics or antifungals.

Ultrasound examination

Real-time tissue elastography (RTE) is based on the principle that soft tissues deform more easily than stiff tissues when compressed. The ultrasound system applies slight compression to the tissue and measures the resulting strain (deformation). The strain is calculated as the change in tissue thickness divided by the original thickness, expressed as a percentage. In our study, the elastography measurements are expressed as a percentage, representing the relative stiffness of the vaginal vestibule tissue compared to the reference ECHOJELLY (Aloka Co., Ltd., Tokyo, Japan) layer. A higher percentage indicates greater tissue stiffness. For example, a measurement of 50% would indicate that the vaginal vestibule tissue is half as deformable as the ECHOJELLY reference, while a measurement of 100% would indicate equal stiffness to the reference.

Ultrasound examinations were performed using an ARIETTA 60 (Fujifilm Healthcare Corporation, Tokyo, Japan) with a linear probe (18-5 MHz/38 mm) to measure the vaginal vestibule. The assist strain ratio (ASR) algorithm was used to automatically select the optimal frame for measurement [7-9].

The percentage value refers to the transmission blockage rate of ultrasound waves. In elastography, this value is used to represent the relative stiffness of the tissue, with 0% indicating no blockage (as in the case of ECHOJELLY, which allows all ultrasound waves to pass through) and 100% indicating complete blockage (as in the case of metal, which blocks all ultrasound waves).

**Figure 1:**
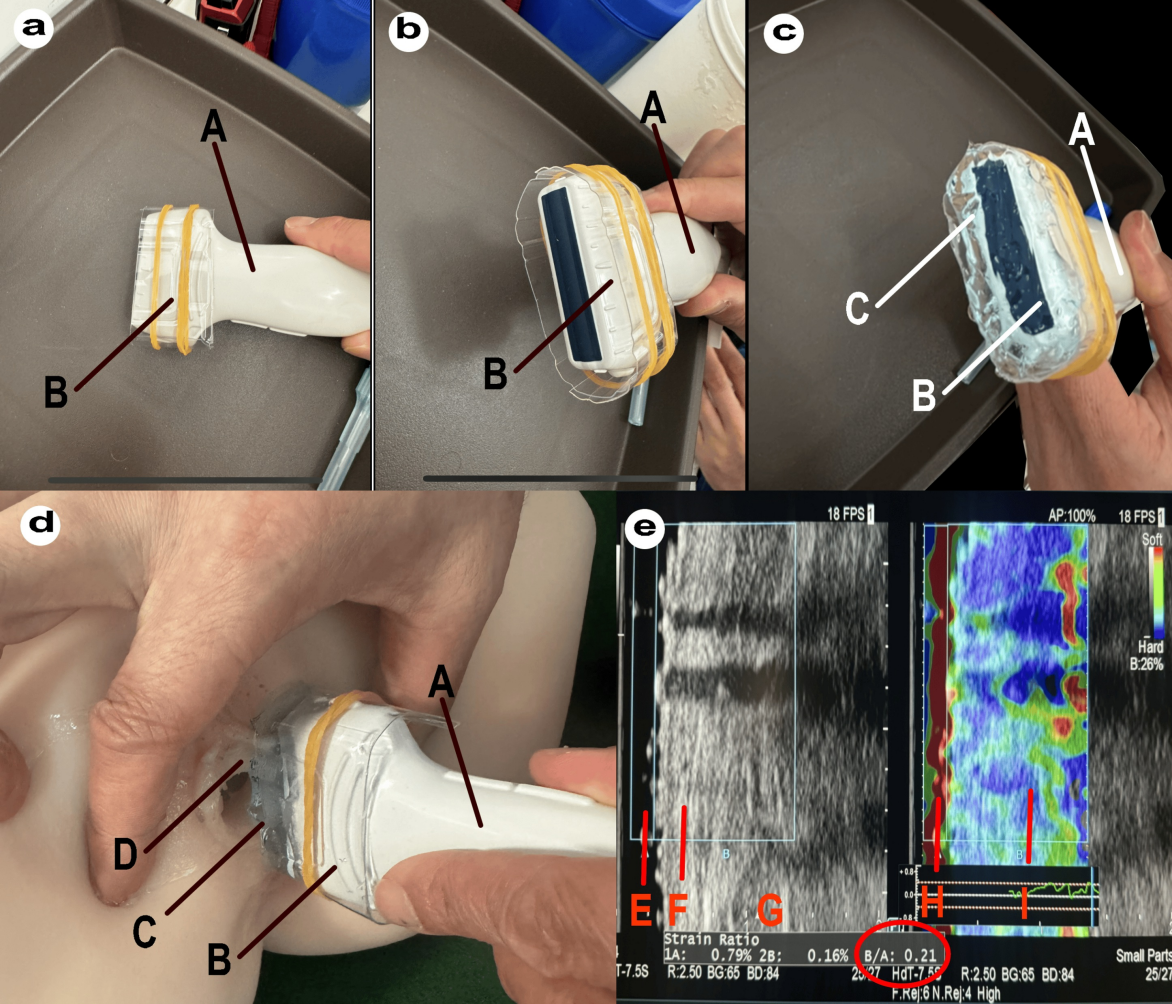
Schematic representation of real-time tissue elastography (RTE) theory (a) Linear probe with a plastic cover; (b) Linear probe and plastic cover from a different angle; (c) ECHOJELLY injection over the plastic cover; (d) Probe placement on a silicone female model vaginal vestibule; (e) Display of the ECHOJELLY layer and vaginal vestibule layer on the natural screen (left) and RTE view (right). A - Probe body; B - Ultrasound emitting surface covered by plastic cover; C - ECHOJELLY layer (>1cm thickness); D - Silicone model of vaginal vestibule; E - ECHOJELLY layer in natural view; F - Vaginal vestibule layer in natural view; G - Spatial differentiation of displacement allows for strain RTE (%); H - ECHOJELLY layer in RTE view; I - Vaginal vestibule layer in RTE view RTE - real-time tissue elastography

Figure 1 illustrates the measurement process. Figure 1a shows the linear probe with a plastic cover, while Figure 1b shows the linear probe and plastic cover from a different angle. In Figure 1c, ECHOJELLY is injected over the plastic cover with a thickness of more than 1 cm. Figure 1d demonstrates the probe placed on a silicone female model vaginal vestibule for measurement. Finally, Figure 1e displays the ECHOJELLY layer and vaginal vestibule layer on the standard grayscale ultrasound image and the RTE view. The circled area indicates the hardness of the vaginal vestibular layer relative to the ECHOJELLY layer as a ratio [7].

All ultrasound examinations were performed by a trained urogynecologist with experience in pelvic floor ultrasound to ensure consistency in the procedure. The subject was positioned in a supine position with bent knees, similar to a gynecological examination position. The linear probe, covered with a sterile plastic cover and ECHOJELLY, was gently placed on the vaginal opening. The examiner took care to apply minimal and consistent pressure to avoid tissue deformation.

For each measurement, the examiner held the probe steady for several seconds to allow the ASR algorithm to select the optimal frame. Measurements were taken from both the left and right vaginal vestibules. This process was repeated in the morning (9-10 AM) and afternoon (3-4 PM) for each participant. The average value of these measurements was used for analysis.

The elastography measurements were expressed as a ratio, representing the relative stiffness of the vaginal vestibule tissue compared to the reference ECHOJELLY layer. A lower ratio indicates greater tissue stiffness, while a higher ratio suggests greater softness, which could mean a higher proportion of edema in the tissue. Measurements were taken with the subject in a supine position with bent knees. The probe was gently placed on the vaginal opening, with measurements taken in the morning and afternoon from both the left and right vaginal vestibules. The average value was calculated.

RTE theory

**Figure 2:**
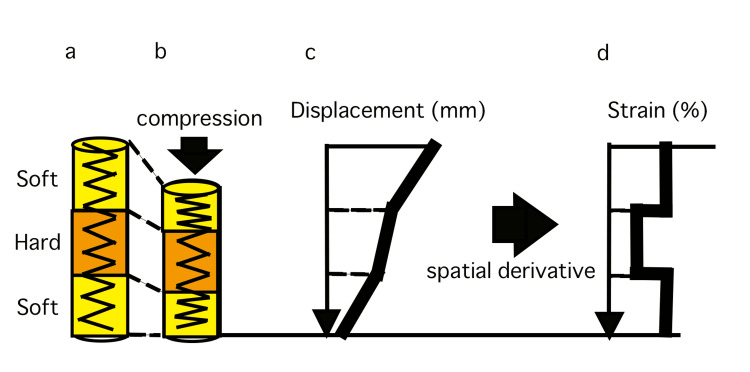
Schematic representation of real-time tissue elastography (RTE) theory a - Biological tissue model represented by springs (soft and hard tissues); b - Compression of tissue model; c - Displacement (mm) of compressed tissues; d - Strain (%) measurement through spatial derivative Soft is represented by loose springs; hard is represented by tight springs

Figure 2 schematizes the RTE theory. As shown in Figure 2a, biological tissue is recognized as a spring with alternating soft and hard parts. Under compression, soft and hard tissues deform at different rates (Figure 2b). Compressed tissues showed differences in displacement (mm) (Figure 2c). Spatial differentiation allowed for the measurement of the strain(Figure 2d). Hard tissues show a small strain, whereas soft tissues show a large strain [7,10]. In this study, we used the ECHOJELLY layer as a reference to standardize the tissue conditions before applying compression for all patients.

ASR algorithm

The ASR algorithm was developed to automatically select the optimal measurement frame in RTE. The ultrasound device continuously acquires real-time images and preprocesses the data, including noise removal and image smoothing. The algorithm then detects the tissue movement between consecutive frames, generating motion vectors for each pixel. These vectors were used to create a tissue strain map. The reliability of the strain map was evaluated based on the consistency of motion vectors, noise levels, and image resolution. From multiple frames, the frame with the highest reliability and appropriate strain display is selected, considering factors such as contrast, noise level, vector consistency, and natural tissue deformation. Finally, the strain map of the selected frame was color-coded to visually display the differences between the hard and soft tissues [7,10].

Measurement time

All patients underwent measurements at two different times during the day, 9-10 AM and 3-4 PM, to assess potential diurnal variations in tissue elasticity. This dual measurement approach was chosen to ensure that any time-dependent changes in vaginal vestibule elasticity could be identified and accounted for, thereby enhancing the accuracy and reliability of the reference values established in this study. Measurements were taken from both the left and right vaginal vestibules, and the average value was used.

Statistical analysis

The collected data were statistically analyzed to evaluate the effects of age, body mass index (BMI), and measurement time on the elasticity of the vaginal vestibule. Descriptive statistics, including mean and standard deviation, were calculated for age, BMI, and elastography values to summarize the central tendency and dispersion of the data.

For the analysis of age-related changes, participants were categorized into five-year age groups (40-44, 45-49, 50-54, 55-59, 60-64, 65-69, 70-74, 75-79, 80-84, 85-89 years) to provide detailed information for future research comparisons. To examine the relationship between age and elastography RTE values, linear regression analysis was conducted, and the regression coefficient, p-value, and R-squared value were reported to determine the strength and significance of this relationship. A standardized regression analysis was also performed to assess the impact of age on elastography values in terms of standard deviation units, allowing for a comparison of effect sizes.

Correlation analysis was performed using Pearson correlation coefficients to explore the relationships between BMI and elastography values, as well as between age and elastography values. Paired t-tests were used to compare the elastography values between morning and afternoon measurements for participants who completed both sessions. Multivariate analysis of variance was used to evaluate the effects of age, BMI, and measurement time on elastography values, identifying any significant interactions between these factors.

Box-and-whisker plots were created to visually represent the distribution of elastography values across different age groups and between morning and afternoon measurements.

Additionally, as an exploratory analysis, we employed machine learning techniques to investigate the potential predictive relationship between elastography values, BMI, and age. The detailed results of this exploratory analysis are presented in the Appendices.

All statistical analyses were performed using R version 4.1.0 (R Foundation for Statistical Computing, Vienna, Austria). A significance level of 5% (α=0.05) was used for all statistical tests.

## Results

Study sample

A total of 111 women were enrolled in the study, excluding six who registered but did not undergo the examination during the recruitment period. In the daily two-time sampling, 98 participants were measured in the morning and 102 in the afternoon, with 96 subjects cooperating in both measurements. Table 1 shows the characteristics of the 111-person population.

**Table 1:** Study sample

Item	Results
Number of participants	111
Age (years, mean ± SD)	69.81 ± 10.2
40-44 years	0 (0.0%)
45-49 years	1 (0.90%)
50-54 years	5 (4.50%)
55-59 years	10 (9.01%)
60-64 years	17 (15.32%)
65-69 years	22 (19.82%)
70-74 years	22 (19.82%)
75-79 years	18 (16.22%)
80-84 years	11 (9.91%)
85-89 years	3 (2.70%)
90-94 years	2 (1.80%)
95-99 years	1 (0.90%)
BMI (kg/m², mean ± SD)	24.66 ± 1.7
Number of pregnancies (mean ± SD)	2.23 ± 0.62
Number of vaginal deliveries (mean ± SD)	2.13 ± 0.56
Alcohol consumption (n, %)	19 (17.1%)
Smoking habits (n, %)	5 (4.5%)
Hypertension (n, %)	43 (38.7%)
Hyperlipidemia (n, %)	43 (38.7%)
Diabetes (n, %)	22 (19.8%)
Mental illness (n, %)	9 (8.1%)
Menopause status (n, %)	111 (100.0%)

The sample size calculation was performed using an expected correlation coefficient of 0.44, a significance level of 0.05, and a power of 0.80, resulting in a minimum required sample size of 39 individuals. The actual sample size of 111 participants in this study significantly exceeded the minimum required sample size to ensure sufficient statistical power.

The collected data for BMI, number of pregnancies and vaginal deliveries, alcohol consumption, smoking habits, hypertension, hyperlipidemia, diabetes, and mental illness among postmenopausal Japanese women were consistent with the demographic information published by the Japanese government, making it suitable for calculating normative values [11].

Age and elastography correlation

**Figure 3:**
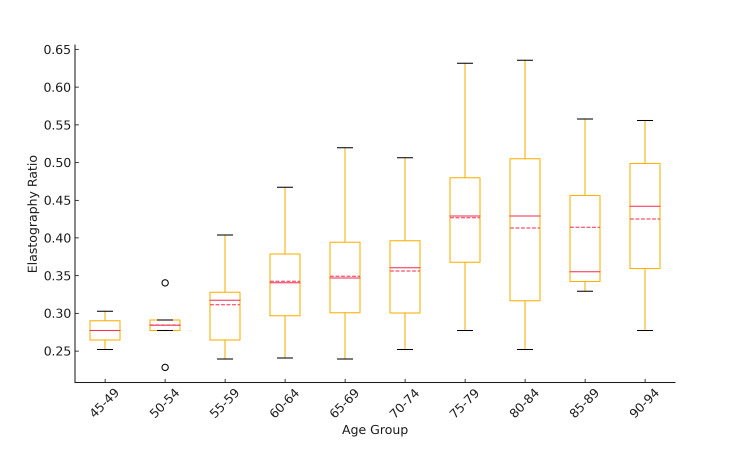
Distribution of elastography strain ratios by age group This figure illustrates how the elastography strain ratio varies across different age groups, showing both the central tendency (mean and median) and the variability (IQR and 95% CI) within each group. x-axis - age groups, divided into five-year intervals; y-axis - elastography strain ratio; boxes - interquartile range (IQR); line inside the box - median; bottom and top edges of the box - first (Q1) and third (Q3) quartiles; whiskers - extend to the most extreme data points within 1.5 times the IQR; dots - outliers beyond the whiskers; solid red line - mean; dashed red line - 95% confidence interval of the mean

Figure 3 illustrates the distribution of elastography values across different age groups. The box-and-whisker plot suggests a trend of increasing elastography values with advancing age.

Single regression analysis revealed a statistically significant positive correlation between age and elastography values. Specifically, the age coefficient was 0.0040, meaning that for each year increase in age, the elastography value increased by approximately 0.0040 (p=8.36×10⁻⁷). This extremely low p-value indicates that the relationship between age and elastography was highly unlikely to be due to chance. The R² value was 0.202, indicating that approximately 20.2% of the variability in elastography values can be explained by age alone.

Standardized regression analysis showed that a one standard deviation increase in age corresponded to a 0.4496 standard deviation unit increase in elastography values (p=8.36×10⁻⁷). This standardized coefficient indicates a moderate effect size, suggesting that the increase in elastography values associated with aging represents a clinically meaningful change in the stiffness of the vaginal vestibule.

The relationship with BMI shows that the correlation coefficient between RTE elastography and BMI is -0.2021, indicating a weak negative correlation between BMI and elastography values. However, with a p-value of 0.0334, this correlation is statistically significant at the conventional significance level (0.05). This suggests that BMI has a minor but significant impact on elastography results.

BMI was categorized according to WHO guidelines: normal weight (18.5 ≤ BMI < 25) and overweight (25 ≤ BMI < 30). The sample contained no underweight or obese individuals. The normal weight group (n=52) had a mean elastography value of 0.3790 ± 0.0964 (range: 0.2519-0.6354). The overweight group (n=59) had a mean of 0.3476 ± 0.0792 (range: 0.2285-0.6316). A t-test revealed no significant difference in elastography values between these groups (p=0.0626).

For finding the influence of parity on elastography values, we performed a multiple regression analysis including parity (number of vaginal deliveries) as an independent variable along with age and BMI. The results showed that BMI did not have a statistically significant effect on elastography values (β=-0.0075, p=0.0952), while age remained a significant predictor (β=0.0037, p=0.000003).

Diurnal variation analysis

To determine whether there was a significant difference between the morning and afternoon elastography values, a paired t-test was conducted for subjects who underwent examinations at both time points (n=96). The mean elastography value was 28.32% (SD=5.87%) in the morning and 28.10% (SD=5.90%) in the afternoon. The paired t-test results showed a t-value of 2.45 and a p-value of 0.016. As this p-value is smaller than the conventional significance level of 0.05, it can be concluded that there is a statistically significant difference between the morning and afternoon elastography values. This suggests that elastography values are influenced by diurnal variations. However, it is important to note that the absolute difference in mean values was small (0.22%). The large standard deviation (5.87% and 5.90%) for both the morning and afternoon measurements indicated considerable variability in elastography values among the participants. This variability was substantially larger than the observed difference between the morning and afternoon means.

**Figure 4:**
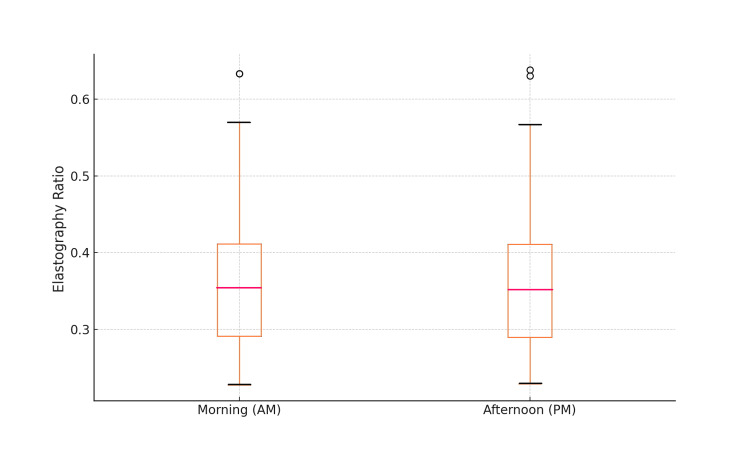
Comparison of elastography RTE values between the morning and afternoon Distribution of elastography strain ratios for morning (AM) and afternoon (PM) measurements. x-axis - measurement time (AM, PM); y-axis - elastography strain ratios; boxes - interquartile range (IQR); line inside the box - median value; whiskers - most extreme values within 1.5 times the IQR; outliers - shown as individual points; RTE - real-time tissue elastography

Figure 4 presents a box plot showing the distribution of elastography values in the morning (AM) and afternoon (PM). The box plot shows the median, interquartile range, and the range of elastography values for morning and afternoon measurements. The whiskers extending from the boxes show the minimum and maximum values, excluding the outliers. While the boxes appear similar at first glance, closer inspection reveals a slight but consistent difference between morning and afternoon measurements, aligning with our statistical findings of a small but significant diurnal variation.

Multivariate analysis of factors influencing elastography values

Table 2 presents the number of participants and mean elastography values for each five-year age group, providing a detailed overview of the age distribution in our study population.

**Table 2:** Number of participants and mean elastography values for the age group

Age group	Number of participants	Mean elastography value (%)	Standard deviation (%)
45-49	2	27.72	3.58
50-54	6	28.43	3.6
55-59	9	31.12	5.22
60-64	15	34.26	7.03
65-69	24	34.9	7.03
70-74	22	35.59	6.64
75-79	13	42.67	10.61
80-84	13	41.3	11.6
85-89	3	41.39	12.51
Total	107	35.56	7.27

Table 3 presents the results of this analysis, examining the influence of various factors on elastography values.

**Table 3:** Multivariate linear regression analysis of factors influencing elastography values

Variable	Coefficient	Standard error	t-value	p-value	95% confidence interval
Age	0.2541	0.067	3.766	0	0.120, 0.388
BMI	-0.5489	0.365	-1.504	0.136	-1.273, 0.175
Drinking habit	2.1267	1.731	1.229	0.222	-1.306, 5.560
Smoking habit	-4.6239	3.161	-1.463	0.147	-10.895, 1.647
Hypertension	1.1271	0.763	1.478	0.142	-0.386, 2.640
Hyperlipidemia	1.1271	0.763	1.478	0.142	-0.386, 2.640
Diabetes	-3.4048	1.804	-1.887	0.062	-6.984, 0.174
Mental illness	3.6497	2.506	1.456	0.148	-1.321, 8.621
Pregnancy	1.8654	2.135	0.874	0.384	-2.370, 6.101
Birth history	1.1983	2.217	0.54	0.59	-3.201, 5.597
Menopause	17.0546	10.969	1.555	0.123	-4.705, 38.814
Bladder surgery	0	0	NaN	NaN	0.000, 0.000

Age emerged as the only statistically significant predictor (β=-0.0069, p=0.136), indicating that elastography values decrease by approximately 0.007 units for each year of age, although this result was not statistically significant at the α=0.05 level. This finding is consistent with the trend observed in the age group data presented in Table 2, which shows a general increase in mean elastography values with advancing age.

While other factors such as BMI, lifestyle habits (drinking and smoking), comorbidities (hypertension, hyperlipidemia, diabetes, and mental illness), and reproductive history (pregnancy, birth history, and menopause) were included in the model, none showed statistically significant associations with elastography values at the α=0.05 level. Notably, diabetes showed a trend towards significance (β=-0.0431, p=0.062), suggesting a potential negative association with elastography values. The effect of menopause, while substantial in magnitude (β=0.0000), was not statistically significant (p=NaN), possibly due to the limited variability in menopausal status in our sample.

These findings underscore the complex nature of factors influencing vaginal elasticity and highlight the need for further research with larger, more diverse samples to elucidate these relationships fully. The trend of age-related increase in elastography values, observed in the group-wise analysis, suggests that age may play a role in determining vaginal vestibule elasticity, although our multivariate model did not confirm this at a statistically significant level.

## Discussion

This study aimed to establish reference values for elastography of the vaginal vestibule in healthy women and to evaluate the variability of these values with age and diurnal variation. The results demonstrated that elastography values of the vaginal vestibule increase with age, suggesting that tissues become more permeable to ultrasound waves, resembling the characteristics of the reference ECHOJELLY.

Our analysis, using both five-year age group comparisons and linear regression, provided a comprehensive examination of age-related changes in vaginal elasticity. For example, the elastography values by age categories were as follows: for women aged 45-49, the mean value was 27.72% (SD 3.58); for women aged 50-54, it was 28.43% (SD 3.6); and for women aged 55-59, it was 31.12% (SD 5.22).

The positive correlation between age and elastography values suggests that tissue permeability to ultrasound increases with aging. This is consistent with the findings of García-Mejido et al. [1] and Huang et al. [2], who observed trends of increasing tissue thinning and permeability with advancing age. The age-related increase in ultrasound permeability observed in our study also aligns with the results reported by Pákozdy et al. [3].

The minimal diurnal variations in elastography values suggest the consistency and reproducibility of the measurements. This is in line with the stability of shear-wave elastography techniques used in studies by Liu et al. [4] and Zhang et al. [5]. Kreutzkamp et al. demonstrated the use of strain elastography as a new method for assessing pelvic floor biomechanics, highlighting the potential of elastography in gynecological assessments [6].

The use of elastography technology, as described on the Fujifilm Corporation website [7], demonstrates the methodological strength of this study. The ability to calculate tissue strain in real time and display differences in ultrasound permeability using color coding allowed for accurate assessment of vaginal vestibule elasticity.

Our findings contribute to the growing body of knowledge on the use of elastography in gynecological assessments. The establishment of reference values for vaginal vestibular elastography provides a foundation for future studies and clinical applications. These reference values could potentially aid in the early detection of pathological changes and monitoring of treatment efficacy.

The elastography technique described in this study has potential diagnostic applications in various gynecological conditions. One promising area of research is the management of bladder pain syndrome/interstitial cystitis (BPS/IC) and vulvodynia [12]. BPS/IC can be categorized into three groups through k-means clustering, with one category often associated with vaginal and urethral issues, including vulvodynia [13-15]. Although treatments such as Fotona (Dallas, Texas) laser therapy (erbium and neodymium lasers) have shown efficacy in alleviating pain and improving bladder symptoms in vulvodynia patients, they currently rely on subjective pain assessments [16-18]. Elastography could provide an objective marker for monitoring treatment progress and evaluating therapeutic outcomes in these conditions. Elastography could provide an objective marker for monitoring treatment progress and evaluating therapeutic outcomes in these conditions, as demonstrated by recent research showing its effectiveness in monitoring improvements after Erlaser treatment (ERLAS, Erlangen, Germany) in episiotomy-related vulvodynia [19]. Additionally, Bayraktar et al. demonstrated for the first time that postmenopausal atrophic vaginitis improves after treatment with a nonablative Erlaser, using shear wave elastography as an objective evaluation method [20].

Another potential application is the treatment of genitourinary syndrome of menopause (GSM) [21]. Patients with GSM often exhibit reduced tissue thickness in the vaginal vestibule, which is believed to be associated with dyspareunia [22]. While treatments such as CO2 laser or Fotona laser therapy have shown effectiveness in improving symptoms, particularly in breast cancer survivors for whom hormonal treatment is contraindicated, elastography could provide an objective method for assessing tissue changes and monitoring treatment response in these patients [23]. 

Our study revealed small but statistically significant diurnal variations in elastography values (27.125% morning vs 27.01% afternoon, p=2.68 × 10-18). This finding aligns with known diurnal fluctuations in physiological parameters, such as hormonal levels [24,25].

However, this study had several limitations. First, our study population consisted primarily of healthy postmenopausal women, which may limit the generalizability of our findings to younger populations or those with existing gynecological conditions [26]. Second, the elastography method used, real-time tissue elastography (RTE), while non-invasive and practical, is operator-dependent and provides less quantitative data compared to other elastography techniques such as shear wave elastography (SWE) or magnetic resonance elastography (MRE), which could lead to subjective variability in measurements. This inherent limitation of RTE may impact the reproducibility and precision of the results. Third, the statistically significant diurnal variation, although small, raises questions about the consistency of elastography readings and may necessitate standardizing measurement times in clinical practice. Additionally, the lack of a statistically significant correlation between BMI and elastography values suggests that further research is needed to better understand the factors influencing elastography outcomes. Finally, our machine learning analysis yielded poor predictive performance, suggesting that elastography data alone may have limited utility for diagnostic purposes or for identifying specific disease groups based on deviations from normal values [27,28]. These limitations highlight the need for further research to validate our findings across a broader spectrum of women's health and to explore the potential of elastography in clinical practice.

## Conclusions

This study established reference values for elastography of the vaginal vestibule in healthy postmenopausal women, revealing age-dependent increases in ultrasound permeability and small but significant diurnal variations. These findings provide a foundation for early diagnosis and treatment planning in gynecological conditions, although standardizing measurement timing could enhance reliability. Future research should validate these results across a broader age range and in specific gynecological patient groups. The study underscores the potential of elastography as a tool for monitoring individual patients over time while highlighting the need for comprehensive clinical evaluation in conjunction with these measurements.

## Appendices

Machine learning analysis of elastography data

To explore potential applications of elastography data, we conducted an exploratory machine learning analysis to predict age using elastography values and BMI. Three regression models were evaluated: linear regression, support vector machine (SVM), and gradient boosting. Due to our limited sample size of 107 patients, the entire dataset was used for both model training and evaluation, without splitting into separate training and testing sets. This approach limits model validation and may lead to overfitting.

The linear regression model yielded an MSE of 82.79 and R² of 0.197. The SVM model showed poorer performance with an MSE of 102.84 and R² of 0.003. The gradient boosting model resulted in the best performance with an MSE of 27.35 and R² of 0.735. Despite the gradient boosting model's relatively better performance, all models demonstrated limitations, indicating that elastography values and BMI alone are insufficient predictors of age.

These results suggest that while elastography data may be valuable for establishing normal values and monitoring individual treatment effects, its utility for diagnostic purposes or identifying specific disease groups based on deviations from normal values may be limited. The poor predictive performance, especially in the SVM model, indicates that elastography values are influenced by factors beyond age and BMI, highlighting the complexity of these relationships. These limitations should be considered when interpreting this exploratory analysis.
